# Longitudinal Relations Between Social Media Use and Cyberbullying Victimization Across Adolescence: Within-Person Effects in a Birth Cohort

**DOI:** 10.1007/s10964-025-02205-9

**Published:** 2025-06-26

**Authors:** Habib Niyaraq Nobakht, Lars Wichstrøm, Silje Steinsbekk

**Affiliations:** 1 Department of Psychology, Norwegian University of Science and Technology (NTNU), Trondheim, Norway; 2 Department of Child and Adolescent Psychiatry, St Olavs Hospital, Trondheim, Norway

**Keywords:** Cyberbullying, Longitudinal research, Online bullying, Social media use, Victimization, Within-Person analysis

## Abstract

Cyberbullying involves aggressive behaviors or threats through digital platforms. Youth who are victims of cyberbullying are at risk for a wide range of emotional and behavioral problems. Given the growing role of social media in adolescent life, understanding its relation to cyberbullying is crucial for prevention and policy. Although numerous studies suggest that social media use predicts cyberbullying victimization, methodological shortcomings limit their ability to infer the etiological role of social media use in cyberbullying victimization at the individual level—an issue this study addresses. A sample from two birth cohorts of children (*n* = 781, 53.4% girls) in Trondheim, Norway, was assessed biennially through interviews and questionnaires from age 12 to 18. Social media use and cyberbullying were related at the between-person level (i.e., those who use social media more than others were more likely to experience cyberbullying than others). However, within-person increases in self- or other-oriented social media use did not predict future within-person changes in cyberbullying victimization. The vast majority of former studies, which have not explored within-person changes, may have overestimated and overinterpreted the role of social media use in cyberbullying victimization. Efforts to reduce cyberbullying victimization by decreasing individual social media use may have limited effectiveness.

## Introduction

Social media may play an important role in adolescent socialization, offering opportunities for social support and identity exploration (Avci et al., 2024, Zhou & Cheng, 2022). Associated with both higher well-being *and* ill-being (Valkenburg et al., 2022), social media use benefits many adolescents while also putting them at risk of online aggression, such as cyberbullying. Cyberbullying refers to bullying through electronic means in a digital space where a perpetrator employs aggressive behaviors or threats, such as verbal abuse and humiliation, against victims (Smith et al., 2008). From 2007 to 2023, the rate of cyberbullying victimization over the past 30 days among U.S. adolescents increased from approximately 10% to 26%, while the lifelong rate increased from 20% to 54% (Patchin & Hinduja, 2023). Cyberbullying victimization most commonly occurs on social media (Kowalski et al., 2019; Seitz, 2024), and two meta-analyses (Fisher et al., 2016; Marciano et al., 2020) found that cyberbullying victimization is associated with a range of internalizing and externalizing problems. For prevention and policy reasons, it is therefore essential to understand the relation between social media use and cyberbullying victimization. To this end, researchers need to distinguish between two key questions: i) Are those who use social media more than others also cyberbullied more than others?—a between-person question, and ii) Does an adolescent who increases their own social media face a heightened risk of being cyberbullied compared to their usual level?—a within-person question. Thus, in this study, four waves of biennially collected data from ages 12 to 18 years in two birth cohorts of Norwegian children were analyzed. Using a within-person approach, it is explored whether increased social media use serves as a risk factor for cyberbullying victimization, allowing for a more precise discussion of its implications for policy, prevention, and intervention.

### Social Media Use and Cyberbullying Victimization

A meta-analysis of data from 42 countries showed that intense and problematic social media use—defined as having online contact almost all the time throughout the day with someone and symptoms of social media addiction—predicted a higher risk of both cyberbullying victimization and perpetration (Craig et al., 2020). Similarly, two reviews (Floros & Mylona, 2022; Kowalski et al., 2019) and a meta-analysis (Marciano et al., 2020) concluded that time spent online, engaging in online social interactions, and general Internet use were associated with cyberbullying victimization both concurrently and longitudinally. However, as has been advocated, different social media behaviors may differently affect the outcomes in question, also for cyberbullying victimization (Orben et al., 2024). Despite this, little research has examined whether certain types of social media use pose a greater risk of cyberbullying victimization than others (Fredrick et al., 2022).

Social media platforms offer different affordances, such as posting (sharing content), liking, and commenting. These affordances enable adolescents to engage in various behaviors, including sharing content and self-presentation (i.e., publicness and visualness; Nesi et al., 2018) and providing social feedback to others. Subsequently, different platforms influence not only what users are exposed to and how they experience social interactions (Orben et al., 2024), but also the varying levels of permanence, publicness, and availability that shape how online behaviors are enacted (Nesi et al., 2018). These may play differential roles in shaping the risk of cyberbullying victimization. Similarly, an active use of social media (e.g., posting, commenting) is more likely to expose one to cyberbullying than passive use (e.g., scrolling, consuming content online). Posting content, such as written posts or photos can be considered self-oriented social media use as it involves actively presenting oneself by sharing opinions, experiences, relationships, possessions, or appearances. In contrast, liking and commenting on others’ posts, which often shift the focus away from oneself; merely liking does not imply much self-presentation (Steinsbekk et al., 2021). In other research where the data from the present cohort are utilized it has been found that other-oriented, but not self-oriented, social media use affected the development of physical appearance self-esteem in adolescent girls (Steinsbekk et al., 2021) but were unrelated to symptoms of anxiety and depression (Steinsbekk et al., 2023). While posting content may expose adolescents to a greater degree of public scrutiny, increasing their risk of negative feedback—including cyberbullying—research suggests that adolescents primarily receive positive reactions to their posts (Cingel et al., 2022). Therefore, it is important to investigate whether specific social media behaviors such as posting versus interacting with others’ content differentially predicts adolescents’ risk of cyberbullying victimization.

## Current Study

Scholars have often interpreted the link between social media use and cyberbullying victimization as evidence that social media use *contributes or leads* to more cyberbullying victimization. However, no previous study has positioned itself to reveal whether an individual adolescent who increases their own level of social media is at heightened risk of experiencing increased cyberbullying victimization—a within-person question. Answering this question can inform conclusions concerning the etiological role of social media use in cyberbullying. Traditional cross-lagged approach blends between- and within-person information, and findings from such approaches do not necessarily conform with, and even can contrast, results obtained from pure within-person analyses. In contrast to Cross-Lagged Panel Model (CLPM), within-person analyses not only portray whether *changes* predict *changes* within the same individual, a key aspect of individual development that is essential when informing the development of intervention programs but also adjust for all time-invariant confounders by design (e.g., socioeconomic status, genetics). In addition to separating within-person relations from between-person associations, this study also explores the differential roles of types of social media use (self- and other-oriented) in cyberbullying victimization. Accordingly, biennially collected data from two cohorts of Norwegian children spanning ages 12 to 18 are analyzed using a cross-lagged within-person approach. Additionally, a traditional CLPM is employed to assess whether previous studies may have overestimated and misinterpreted the etiological role of social media use in cyberbullying victimization.

## Methods

### Participants and Procedure

The present inquiry uses data from the longitudinal birth cohort Trondheim Early Secure Study (TESS; Wichstrøm et al., 2012). All children born in 2003 and 2004 (*N* = 3456) in Trondheim, Norway, and their parents were invited to participate in the TESS. Each family received an invitation letter and a copy of the Norwegian version of the Strengths and Difficulties Questionnaire (SDQ; Goodman et al., 2000), which was mailed to their homes. Parents were instructed to complete the SDQ and bring it to their 4-year-olds’ mandatory health checkup at their local well-child clinic. Of the 3456 invited families, 3358 attended the clinic. Among them, 176 families were excluded due to limited proficiency in Norwegian, and 166 families were missed being asked to participate by the health nurse. This left 3016 families, of whom 2477 (82.1%) consented to participate. To enhance statistical power, children with emotional or behavioral problems were oversampled (due to the initial aim of the overarching project being the study of mental health), which was accounted for in the analyses. To achieve oversampling, the children were grouped into four strata based on their SDQ scores (cut-offs: 0–4, 5–8, 9–11, 12–40), with higher scores increasing the probability of selection (0.37, 0.48, 0.70, and 0.89 in four strata, respectively). Subsequently, a final sample of 1250 families was drawn. The study was approved by the Regional Committee for Medical and Health Research Ethics (Steinsbekk & Wichstrøm, 2018).

A total of 1007 families met for the first assessment at the university clinic (T1; age 4), and they were assessed biennially until the child reached age 18. Social media was assessed from age 10 onwards, and cyberbullying at ages 14, 16, and 18 years. To maximize the use of available data, social media use data from ages 12 to 18 and cyberbullying victimization data from ages 14 to 18 were included. The analytical sample, containing at least one data unit on any of the variables, comprised 781 participants (53.4% girls). Participant sex was determined using their national ID number, which reflects their sex assigned at birth. Data collection at T7 (age 16) overlapped with the COVID-19 pandemic restrictions. While the study protocol and measurement instruments remained unchanged, data collection was stopped from March to May 2020 and in January and February 2021, hence delayed by 4.5 months at T7 (*M*_*age*_ = 16.98 years) due to a short stop in data collection during the most severe restrictions and delays due to infections among participants and their parents.

Attrition analyses showed that more other-oriented social media use (i.e., liking and commenting on others’ social media posts) at age 14 predicted higher retention two years later (OR = 1.01, *p* = 0.041); less cyberbullying victimization and self-oriented social media use (i.e., posting updates and photographs on own platform) at age 16 predicted higher retention at age 18 (cyberbullying victimization: OR = 0.34, *p* = 0.026; self-oriented social media: OR = 0.99, *p* = 0.034)). Even though some bivariate associations with missingness were identified as significant (0.01 < *p* < 0.05), an overall test of missingness using the Little’s Missingness Completely at Random (MCAR) test which take into account the number of predictions examined, showed that the attrition was missing completely at random (*X*^*2*^ = 228.09, df = 206, *p* = 0.14).

### Measures

#### Social media use

Social media use was measured by interviews conducted by trained personnel. The main focus of the present study was on active use of social media (i.e., self- and other-oriented) rather than passive use (e.g., scrolling, consuming content online). The measure contained the total monthly sum of liking, commenting, and posting, which captured the participants’ responses to the following questions: 1)‘How often do you like others’ updates?‘; 2) ‘How often do you write comments on others’ updates or photos?‘; 3) ‘How often do you post (written) updates on your own social media sites/accounts?‘; 4) ‘How often do you post photos?’. At ages 16 and 18, the participants were asked to specify the type of photos they posted by two questions: ‘How often do you post photos that are selfies?‘ and ‘How often do you post photos that are not selfies?‘. The aim was to differentiate between these two and specifically study selfie behavior in the future. However, the sum of posting (i.e., regardless of type of photo) should be comparable to the responses to the general question asked at earlier time points (i.e., ‘How often do you post photos’). No questions were specific to certain social media platforms, but as the participants were interviewed, the interviewers would offer examples of social media sites if needed or assist in accurate recalling in other ways (e.g., ’If you think about this week…’). *Self-oriented social media use* constitutes frequency of posting written posts or photos (including the item on selfies assessed from age 16 onwards), whereas *other-oriented social media use* was measured by questions on liking, and commenting on, others’ posts.

The interviewer asked the same questions at all time points, and participants reported frequency of the specific behaviors (e.g., age 12: ‘How often do you post photos?’: 1 = ‘every day’; 2 = ‘2–6 times a week’; 3 =‘once a week‘; 4 = ‘3 times per month’; 5 = ‘more seldom/never’). To ensure that what was expected to be an increase in social media use by age and period was covered, the scale was expanded by applying response categories capturing higher frequencies of the behaviors at older ages. At age 16, a 10-point scale was used (1 =’seldom or never’; 2 = ‘1–3 times a month’; 3 = ‘1 time a week’; 4 = ‘2–6 times a week’; 5 = ‘1 time a day’; 6 = 2–5 times a day’; 7 = ‘6–15 times a day; 8 = ‘16–30 times a day’; 9 = ‘31–60 times a day’; 10 = ‘More than 60 times a day’). When calculating self- and other-oriented behaviors, the response categories were recoded so that they were aligned across time points. Higher numbers indicate higher frequency for all social media behaviors captured.

#### Cyberbullying victimization

Cyberbullying victimization was measured by the Norwegian version of the Cyber-Bullying and Victimization Experiences Questionnaire-Greek (CBVEQ-G; Antoniadou et al., 2016). In the present version, only the 12 items measuring cyberbullying victimization were included, while items relating to cyberbullying perpetration and 6 follow-up questions were omitted. Participants were asked to indicate how frequently during the past 3 months they were experiencing cyberbullying (e.g., ‘Has anybody sent you a message (via cell phone or the Internet) in order to mock you, or talk badly to you?’; ‘Has anybody sent you a message (via cell phone or the Internet) in order to threaten you?’). Responses were recorded on a scale from 1 (never) to 5 (every day). The mean of all items was used as an indicator of cyberbullying victimization. The scale demonstrated good internal consistency across all three timepoints (*α* ranging from 0.83 to 0.87).

### Analysis Plan

A Random Intercept Cross-Lagged Panel Model (RI-CLPM; Hamaker et al., 2015) was employed, consisting of the following components: A random intercept for each of the three study variables (i.e., cyberbullying victimization, and self- or other-oriented social media use, respectively) loading on every observed score at each time point with the factor loading set to 1. These random intercepts represent the average of the scores in a variable across development and thus reflect the between-person information. A latent variable was created for each observed variable, which was assigned a loading of 1, and the error variance in the observed variable was set to 0. This approach transfers the variance in the observed variable to its latent counterpart. These latent variables reflect within-person changes (increase or decrease from an individual’s mean value) at each time point. Concurrent correlations were also allowed between the residuals of these latent changes. Finally, the latent changes in each variable (e.g., cyberbullying victimization) were regressed on the latent changes in the same variable (e.g., cyberbullying victimization) and other variables (e.g., self- and other-oriented social media use) at the preceding time point. This approach treats individuals as their own control and allows for examination of both autoregressive and cross-lagged effects. Additionally, a traditional CLPM was conducted, in which each variable at a given time point was regressed on its own prior value as well as on all variables in the preceding time point while allowing correlations among variables within the same time point.

In both models, freely estimated models were explored allowing the paths to differ between lags. To examine if the strength of the significant cross-lagged relations differed by age, Sattora-Bentler scaled chi-square test was used (Bryant & Satorra, 2012). This test determines whether the model fit deteriorated when these cross-lagged paths were constrained to be equal across time points. If the fit does not deteriorate, this indicates that age differences do not exist. When the chi-square comparison test yields nonsignificant differences, the parsimonious model (i.e., the one with more degrees of freedom) should be preferred (Werner & Schermelleh-Engel, 2010). All analyses were conducted using Mplus 8.5 (Muthén & Muthén, 1998–2017). Probability weights were included to account for the overrepresentation of children with emotional and behavioral problems and to achieve corrected population estimates. A robust Maximum Likelihood Estimator (MLR) and a Full Information Maximum Likelihood (FIML) procedure was employed, ensuring that all available data was used.

## Results

Descriptive statistics for the study variables are presented in Table 1. Correlations between the study variables are presented in Table 2. Even though cyberbullying victimization seems to have remained relatively stable across time points, linear growth curve showed that there is in fact a significant decrease in cyberbullying victimization over time (*intercept* = 1.087, *yearly change* = −0.009, *SE* = 0.002, *p* < 0.001). Substantial fluctuations are noticed in reported social media use across time points. Notably, self-oriented use increased sharply at T7, before dropping again at T8 to a level comparable with earlier waves. In contrast, other-oriented use also rose markedly at T7 but remained elevated at T8. It is possible that level of cyberbullying victimization and social media use may have been influenced by the changes associated with the pandemic. However, such changes in levels may not necessarily translate into changes in the associations between social media use and cyberbullying.

**Table 1:** Sample Description and Mean and Standard Deviation for Scores on Social Media Use and Cyberbullying Aggression (*n* = 781)

Timepoint	T5	T6	T7	T8
Nominal Age	12	14	16	18
Mean of Age	12.49 (0.15)	14.35 (0.16)	16.98^a^ (0.31)	18.60 (0.25)
*N*	661	626	638	594
Self-oriented Social Media Use	19.56 (77.23)	18.32 (31.98)	74.45 (341.89)	16.76 (123.16)
Other-oriented Social Media Use	40.05 (36.60)	60.65 (36.08)	510.36 (627.42)	484.44 (619.18)
Cyberbullying Victimization	*n.a*.	1.09 (0.21)	1.07 (0.18)	1.05 (0.16)

The values within parentheses represent standard deviation

*n.a*. not assessed

^a^Data collection for age 16 was delayed due to the COVID-19 restrictions

**Table 2:** Correlations between Self-Oriented and Other-Oriented Social Media Use (SMU) and Cyberbullying Victimization (CV) (*n* = 781)

Variable	1	2	3	4	5	6	7	8	9	10
1. Self-oriented SMU at T5	—									
2. Self-oriented SMU at T6	0.106	—								
3. Self-oriented SMU at T7	0.011	0.014	—							
4. Self-oriented SMU at T8	−0.005	−0.043*	−0.006	—						
5. Other-oriented SMU at T5	0.08***	0.162***	0.120**	0.093***	—					
6. Other-oriented SMU at T6	0.11***	0.274***	0.060	0.006	0.475***	—				
7. Other-oriented SMU at T7	−0.019	0.079	0.163**	−0.029	0.159**	0.201***	—			
8. Other-oriented SMU at T8	−0.014	0.050	0.076	−0.039	0.172***	0.201***	0.435***	—		
9. CV at T6	0.094	0.163***	0.048	−0.009	0.140**	0.164**	0.072	0.006	—	
10. CV at T7	0.052	0.115*	0.009	−0.005	0.115*	0.154***	0.126*	0.040	0.350***	—
11. CV at T8	0.128	0.127	0.030	−0.003	0.026	0.041	0.082	−0.016	0.314*	0.393**

Note *p <.05, **p <.01, ***p <.001

**Fig. 1:**
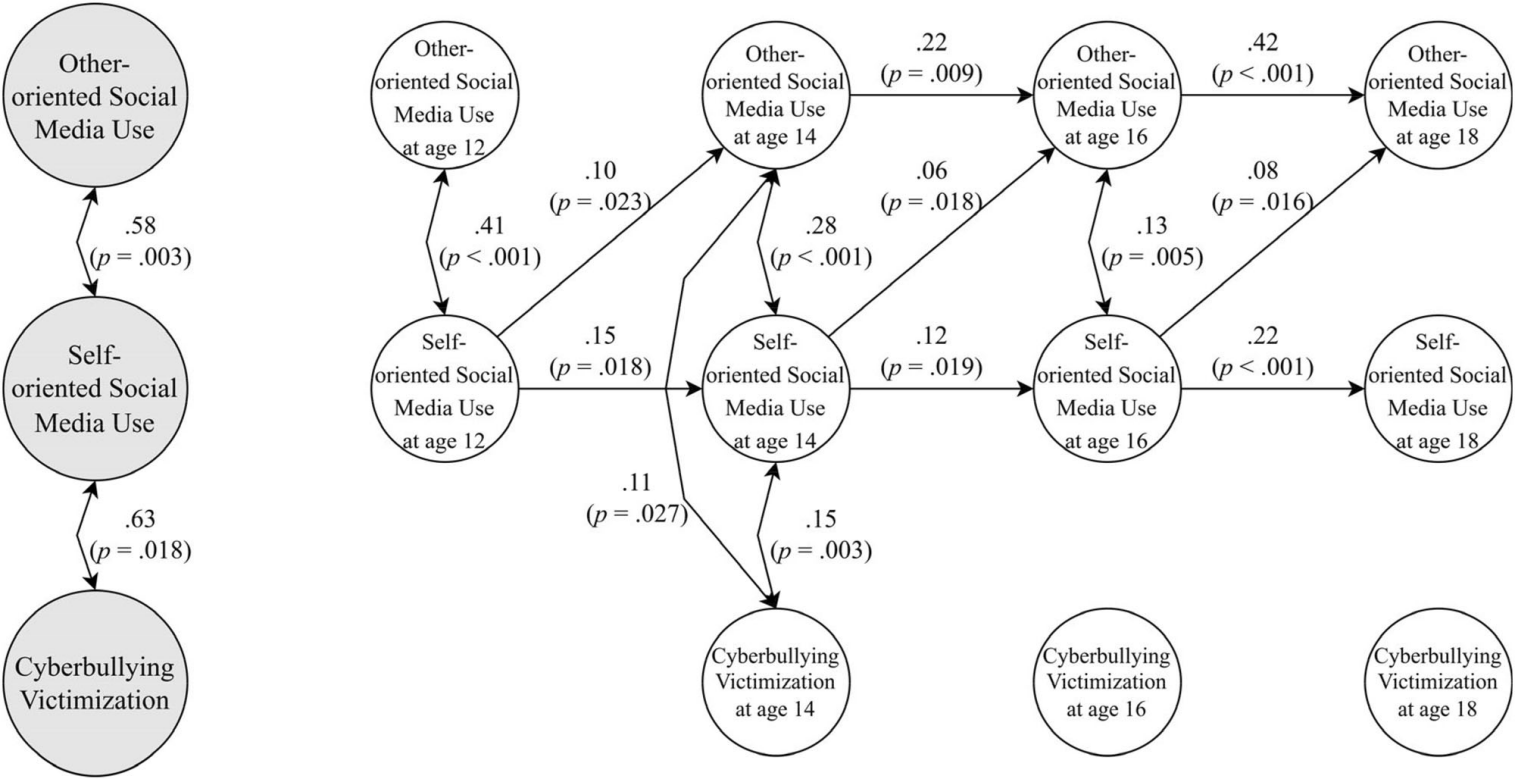
Relations between Self- and Other-oriented Social Media Use and Cyberbullying Victimization from age 12 to 18 in RI-CLPM (*n* = 781). *Note*. Random intercept and within-person estimates are displayed in bigger gray circles and smaller circles, respectively. Only standardized significant paths (*p* < 0.05) are displayed. Paths from random intercepts to their respective latent scores at each timepoint are significant as well but are not depicted here to simplify the figure

Due to the data’s high variance, skewness, and kurtosis, a natural logarithm was applied to all variables, and the transformed data was used in the subsequent analyses. The freely estimated RI-CLPM of the relation between self- and other-oriented social media use and cyberbullying victimization showed excellent model fit (*χ*^*2*^ (15) = 13.09, *p* = 0.595, RMSEA < 0.001, SRMR = 0.020, CFI = 1.00, TLI = 1.00). Only two significant cross-lagged paths were found; those who displayed increased other-oriented social media use at age 12, compared to their mean level, reported increased cyberbullying victimization at age 14 (*β* = 0.14, *p* = 0.014) and those who displayed increased self-oriented social media use at age 14 reported increased other-oriented social media use at age 16 (*β* = 0.15, *p* = 0.027). To examine if the strength of these two paths was significantly different compared to the other age spans, cross-lagged paths from other-oriented social media to cyberbullying victimization, as well as from self-oriented social media use to other-oriented social media use, were constrained to be of equal strength across age. The results revealed that constraining did not deteriorate the model fit (*χ*^*2*^ (19) = 19.90, *p* = 0.401, RMSEA = 0.008, SRMR = 0.024, CFI = 0.999, TLI = 0.996, Δ*X*^2^ (Δ*df* = 4) = 6.99, *p* = 0.136), suggesting that no age differences exist. For parsimonious reasons, the constrained model was preferred, and the estimates are displayed in Fig. 1. In this constrained model, the paths from changes in other-oriented social media use to changes in cyberbullying victimization were nonsignificant (*β* ranging from 0.02 to 0.05, *p* ranging from 0.526 to 0.550). At the between-person level, self-oriented social media use correlated highly with cyberbullying victimization (*r* = 0.64, *p* = 0.018) and other-oriented social media use (*r* = 0.58, *p* = 0.003), indicating that at the group level, these factors are indeed related.

A multigroup model aiming to explore sex differences would not converge. However, when RI-CLPM models were explored for boys and girls separately, the freely estimated RI-CLPM among boys showed an excellent model fit (*χ*^*2*^ (15) = 11.62, *p* = 0.707, RMSEA < 0.001, SRMR = 0.024, CFI = 1.00, TLI = 1.00). None of the within-person prospective cross-lagged paths were significant. At the stable between-person level, self-oriented social media use correlated with cyberbullying victimization (*r* = 0.75, *p* = 0.011). A separate RI-CLPM among girls would not converge and could hence not be estimated. Due to these issues, sex differences could not be investigated.

**Fig. 2:**
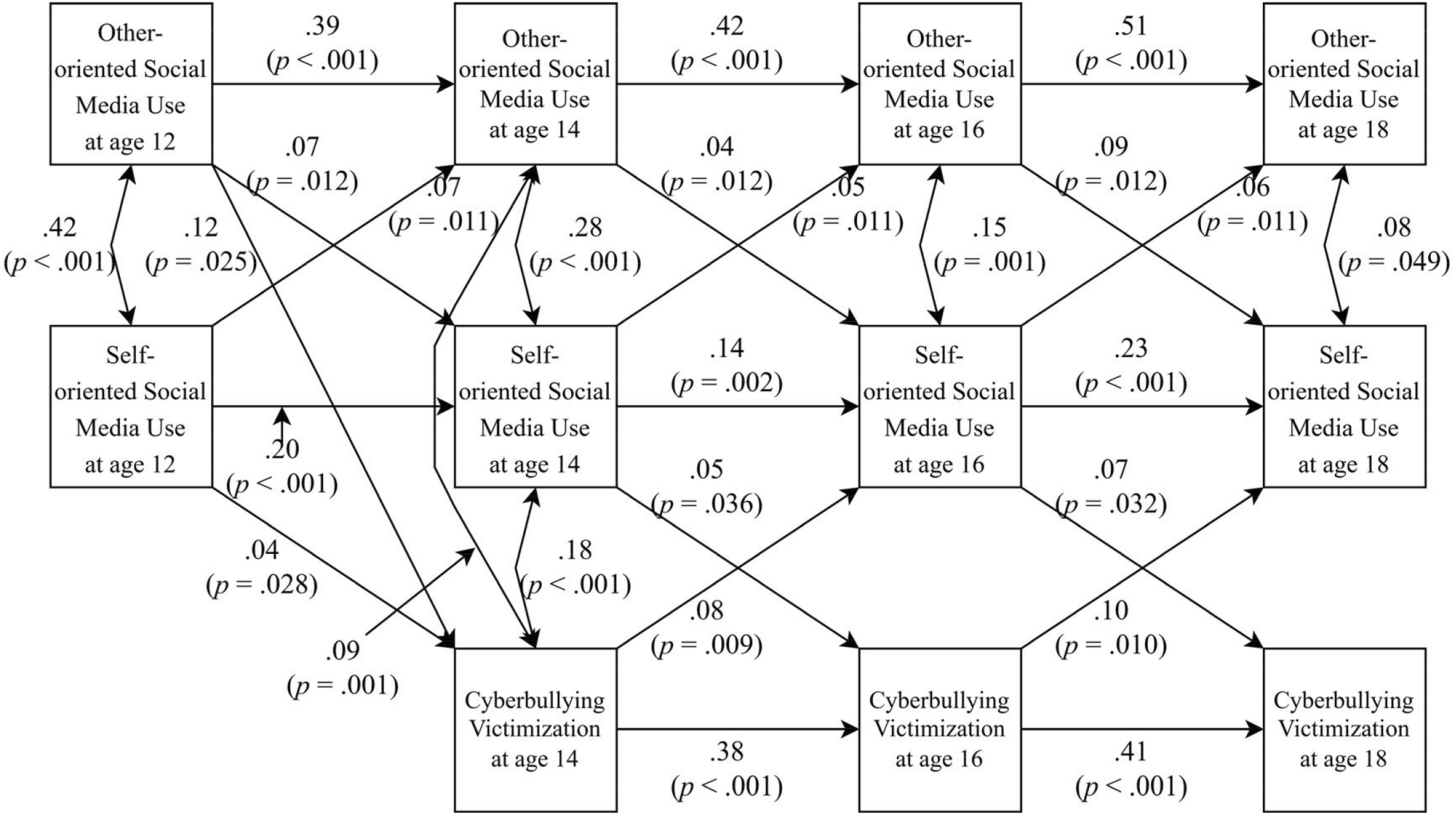
Relations between Self- and Other-oriented Social Media Use and Cyberbullying Victimization from age 12 to 18 in traditional CLPM (*n* = 781). *Note*. Only standardized significant paths (*p* < 0.05) are displayed

To better position the findings from RI-CLPM in comparison with previous studies, a traditional CLPM—which does not separate within- from between-person differences—was estimated. The freely estimated CLPM of the relation between self- and other-oriented social media use and cyberbullying victimization, respectively, showed a good model fit (*χ*^*2*^ (21) = 35.23, *p* = 0.027, RMSEA = 0.029, SRMR = 0.038, CFI = 0.973, TLI = 0.931). Four significant cross-lagged paths were found; cyberbullying victimization at age 16 was predicted by self-oriented social media use at age 14 (*β* = 0.11, *p* = 0.009); self-oriented social media use at age 16 was predicted by cyberbullying victimization at age 14 (*β* = 0.13, *p* = 0.027); and self-oriented social media use at age 14 was predicted by other-oriented social media use at age 12 (*β* = 0.14, *p* = 0.007), and other-oriented social media use at age 16 was predicted by self-oriented social media use at age 14 (*β* = 0.09, *p* = 0.046). To examine if these significant cross-lagged paths differed across development, the four groups of paths (e.g., cyberbullying victimization regressed on self-oriented social media use at each time point) were constrained to be of equal magnitude. These paths could be constrained to be equal without significantly worsening the model fit (*χ*^*2*^ (28) = 46.15, *p* = 0.017, RMSEA = 0.029, SRMR = 0.043, CFI = 0.966, TLI = 0.934, Δ*X*^2^ (Δ*df* = 7) = 10.86, *p* = 0.145). Thus, for parsimonious reasons, this constrained model was preferred. As displayed in Fig. 2, in this model, a bidirectional relation between self-oriented social media use and cyberbullying victimization emerged over time. Specifically, self-oriented social media use predicted cyberbullying victimization (*β* ranging from 0.04 to 0.07, *p* ranging from 0.028 to 0.036), and cyberbullying victimization predicted self-oriented social media use (*β* ranging from 0.08 to 0.10, *p* ranging from 0.009 to 0.010).

## Discussion

Social media platforms are one of the primary spaces where cyberbullying occurs. Therefore, understanding the relation between social media use and cyberbullying is crucial for developing effective preventive measures. Previous studies have not examined whether the link between social media use and cyberbullying victimization occurs at between-person level or at within-person level. The latter would suggest that changes in an individual’s social media use over time predict changes in their cybervictimization. This distinction is explored using data from a large birth cohort sample and found no evidence supporting the assumption that individuals who increase their social media use—be it self- or other-oriented use—are at higher risk for cyberbullying victimization than they typically would be (i.e., using the individual as his/her own control). Consistent with meta-analytical findings (Craig et al., 2020; Marciano et al., 2020), the results of the present study show that social media use correlated with cyberbullying at the group level (i.e., between-person effects), and when using traditional CLPM (i.e., not separating between- from within-person effects), more self-oriented social media use predicted more cyberbullying. This study adds to existing between-person findings by examining two different social media behavior, showing that only self-oriented (i.e., posting updates and photos), while not other-oriented (i.e., liking and commenting) social media use is linked to cyberbullying victimization in approaches that do not filter out between-person relations.

Finding self-oriented, but not other-oriented, social media use to correlate with cyberbullying victimization supports the argument that different social media affordances (e.g., posting, liking, commenting) provide adolescents with different possibilities and expose them to different risks (Orben et al., 2024). These results show that those who engage more in sharing content, such as written posts or photos, are more likely to experience cyberbullying victimization compared to others, reinforcing the notion of Nesi et al. (2018) that certain features such as high permanence, publicness, and visualness may enact different online behaviors (i.e., higher risk of victimization by others). In contrast, liking and commenting were unrelated to cyberbullying victimization, possibly due to the difference in exposure these social media behaviors entail (e.g., less visualness, no enactment of cyberbullying). Importantly, at the within-person level, those who increase neither their self-oriented nor their other-oriented social media use exhibit a higher risk of cyberbullying victimization in the future.

Finding that the between-person relation between self-oriented, but not other-oriented, social media and cyberbullying victimization is significant point to the potential role of common time-invariant factors affecting both social media use and cyberbullying. One likely candidate is genetic predisposition, which is found to explain substantial variance in social media use ranging from around one-third in adolescence (Ayorech et al., 2017) to one-fifth to half in young adulthood (Ayorech et al., 2023). Unfortunately, corresponding studies have not been conducted for cyberbullying, but heritability of victimization from offline bullying has been found to be 65% (Veldkamp et al., 2019). The large Australian twin study 25Up (Mitchell et al., 2019) holds potential to explore the common genetic factors underlying both social media use and cyberbullying victimization when their data is collected over the next years. In addition to genetic factors, personality traits, particularly extraversion and openness, which predict both social media use (Gil de Zúñiga et al., 2017) and cyberbullying victimization (Peluchette et al., 2015), may explain the revealed between-person relations.

Even though cyberbullying victimization showed a significant declining trajectory from age 14 to 18 and the level of cyberbullying victimization predicts its own level across time (see Fig. 2), within-person changes (e.g., decrease) in cyberbullying victimization did not predict future changes (e.g., decrease) in cyberbullying victimization (see Fig. 1). This means that adolescents whose cyberbullying victimization increases at one point relative to their own mean level are not significantly more likely to have been cyberbullied more than they usually are two years later.

The most important finding of this study is that, at the within-person level, neither increases in self-oriented nor other-oriented social media use predict subsequent increases in cyberbullying victimization. This is notable, as previous studies have not explored within-person links and have predominantly relied on analytic methods, such as traditional CLPM, that conflate between- and within-person information. Consistent with this, a traditional CLPM was applied to the current data to compare its results to the results from RI-CLPM. In CLPM, social media use, in particular self-oriented social media use, *did* predict later cyberbullying victimization—confirming prior findings. However, CLPMs are rarely appropriate for inferring causal links about within-person dynamics (Lucas, 2023) and may yield conclusions different from many real-world scenarios in psychology, which occur at the within-person level. Overreliance on such models likely contributes to overinterpretations regarding the impact of social media use on cyberbullying victimization. In contrast, employing RI-CLPM, helps single out within-person effects and controls for all unobserved time-invariant confounding effects by design, such as stable effects of gender, socioeconomic status, or personality traits, by allowing each participant to serve as their own control. Therefore, the results of RI-CLPM in this study provide more robust evidence and suggest that the link between social media use and cyberbullying victimization exists at the between-person level but provide no support for it operating at the within-person level.

### Implications

The lack of support for significant within-person longitudinal relations between social media use and cyberbullying victimization suggests that efforts to reduce social media use—at least as measured by self- and other-oriented social media use—in adolescents may have limited effectiveness in reducing the risk of cyberbullying victimization. Platform policies, including technological interventions using AI and machine learning to detect and remove online abusive communication, could potentially help reduce cyberbullying among social media users (Nee et al., 2023). However, the efficacy of these methods remains limited and questionable (Ademiluyi et al., 2022). State policies to combat negative effects of social media use on adolescents are exemplified by the recent laws passed in several countries imposing an age limit for social media use. These laws may reduce the risk of cyberbullying victimization on social media, at least for those below the age limit. School interventions can focus on implementing anti-bullying policies and expanding anti-bullying materials in school programs to cover cyberbullying. School-based recommended efforts such as teaching digital citizenship skills (Fredrick et al., 2025) can help increase students’ awareness regarding risks of social media use and cyberbullying, preparing students for better digital well-being and healthy online interactions (Smith et al., 2008). Ultimately, a combination of interventions such as technological interventions, state regulations on social media platforms, and school interventions may result in safe social media use and reduced cyberbullying victimization.

### Limitations

Although this research has many strengths, such as longitudinally exploring the within-person relations between social media use and cyberbullying victimization, it also has some limitations. The measurement was limited in capturing only an active use of social media (e.g., posting, commenting), missing other aspects of social media use such as passive use and time spent online. Additionally, here two-year longitudinal effects were examined, thus missing potential shorter-term within-person effects that dissipate over time. As a result, it cannot be determined whether changes in social media use may have predicted changes in cyberbullying victimization if the assessment intervals were shorter (e.g., weekly, monthly). On the other hand, research has shown that, at the within-person level, changes in gaming disorder symptoms and cyberbullying victimization do not predict each other even when assessment intervals are 6 months (Nie et al., 2024). This finding, along with the current results, suggest that changes in an individual’s frequency of using different forms of technology, including social media, do not predict changes in the same individual’s frequency of cyberbullying victimization, regardless of assessment intervals.

## Conclusion

When separating between-person and within-person effects, it becomes evident that the link between social media use and cyberbullying victimization identified in previous research is likely due to a between-persons effect—those who use social media more in a self-oriented way are bullied online more than others. However, compared to their typical mean level, adolescents who *increase* their self- or other-oriented social media use are not at increased risk of becoming *more* bullied online. This does not preclude the possibility that other dimensions of social media engagement (e.g., time spent online, interacting, and chatting) could predict cyberbullying victimization—an important direction for future research. Nevertheless, the findings of this study suggest that efforts to reduce self- or other-oriented social media use at the individual level may have limited effectiveness in reducing the risk of cyberbullying victimization. The between-person associations between self-oriented social media use and cyberbullying victimization highlight the need for changes in policies at the platform, state, and school levels to target cyberbullying while promoting healthy social media use.
